# Zinc recovery from bioleachate using a microbial electrolysis cell and comparison with selective precipitation

**DOI:** 10.3389/fmicb.2023.1238853

**Published:** 2023-08-17

**Authors:** Sabine Spiess, Jiri Kucera, Tomas Vaculovic, Ludwig Birklbauer, Clemens Habermaier, Amaia Sasiain Conde, Martin Mandl, Marianne Haberbauer

**Affiliations:** ^1^K1-MET GmbH, Linz, Austria; ^2^Department of Biochemistry, Faculty of Science, Masaryk University, Brno, Czechia; ^3^Department of Chemistry, Faculty of Science, Masaryk University, Brno, Czechia; ^4^Voestalpine Stahl GmbH, Linz, Austria

**Keywords:** microbial electrolysis cell, metal recovery, zinc recovery, bioleaching, selective precipitation

## Abstract

Metal recycling is essential for strengthening a circular economy. Microbial leaching (bioleaching) is an economical and environmentally friendly technology widely used to extract metals from insoluble ores or secondary resources such as dust, ashes, and slags. On the other hand, microbial electrolysis cells (MECs) would offer an energy-efficient application for recovering valuable metals from an aqueous solution. In this study, we investigated a MEC for Zn recovery from metal-laden bioleachate for the first time by applying a constant potential of −100 mV vs. Ag/AgCl (3 M NaCl) on a synthetic wastewater-treating bioanode. Zn was deposited onto the cathode surface with a recovery efficiency of 41 ± 13% and an energy consumption of 2.55 kWh kg^−1^. For comparison, Zn recovery from zinc sulfate solution resulted in a Zn recovery efficiency of 100 ± 0% and an energy consumption of 0.70 kWh kg^−1^. Furthermore, selective metal precipitation of the bioleachate was performed. Individual metals were almost completely precipitated from the bioleachate at pH 5 (Al), pH 7 (Zn and Fe), and pH 9 (Mg and Mn).

## Graphical abstract

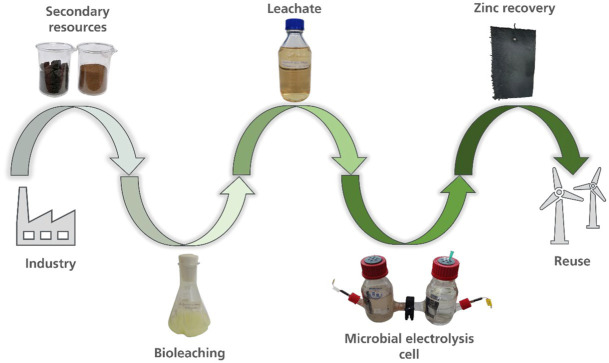

## Introduction

The European Green Deal is proposed as the EU’s strategy to achieve climate neutrality. One key point is decoupling economic growth from resource use and shifting to a circular economy. Therefore, a circular economy of production processes is of high importance (European Commission, 2019). For example, by-products such as blast furnace dust are generated in the steel industry, containing valuable heavy metals such as zinc (Zn). Zn is an essential trace element for microorganisms, animals, humans, and plants. Zn is also the fourth most used metal in the world (e.g., in the galvanizing process) and plays a critical role in renewable energy technologies by preventing solar panels or wind turbines from rusting (International Zinc Association, 2022). Therefore, the recycling of Zn is in high demand to meet the Green Deal goals to minimize CO_2_ emissions and strengthen a circular economy.

In particular, biohydrometallurgical approaches that use microbes to extract metals from waste are becoming increasingly popular. Earlier bioleaching applications on primary sulfide ores are now being applied to wastes. Efficient solubilization of metals, such as iron (Fe), copper (Cu), or Zn, from secondary resources (e.g., waste incineration ashes and slags) by microbial activity has been recently reported (Kremser et al., 2021). Furthermore, the bioleaching of e-wastes, such as printed circuit boards (Yang et al., 2017; Utimura et al., 2019; Andrade et al., 2022) has been intensively investigated. The bioleaching of metal oxides is mainly accomplished by bacterial sulfuric acid production, Fe^3+^ regeneration, and the secretion of complexing agents (Kremser et al., 2020). Still, a crucial step after bioleaching is the metal recovery from the bioleachate.

Various metal recovery methods, such as biosorption, selective precipitation, electrowinning, or bioelectrochemical systems, are currently under investigation (Işıldar et al., 2019). Bioelectrochemical systems are an environmentally friendly platform technology for various applications such as wastewater treatment (Liu et al., 2004), hydrogen production (Hasibar et al., 2020), or the capture and conversion of CO_2_ into valuable products (Spiess et al., 2022).

Electroactive microorganisms employed in the anode chamber of bioelectrochemical systems can oxidize organic sources such as wastewater while generating an electrical current that can drive, in whole or in part (depending on the reduction potential), metal reduction at the cathode. Various metals such as chromium (Cr), cobalt (Co), nickel (Ni), Cu, or Zn have been investigated for their removal and recovery from metal-containing waste streams due to their potential toxic and carcinogen effects and to avoid environmental contamination (Nancharaiah et al., 2015). For instance, Cu (+0.34 V vs. SHE) was recovered at high rates with a microbial fuel cell (MFC) by concomitantly generating a current density of 23 A m^−2^ (Motos et al., 2015). On the other hand, to recover Zn (−0.76 V vs. SHE), a potential must be applied at microbial electrolysis cells (MEC) to enable cathodic reactions (Modin et al., 2017).

However, MECs offer several advantages compared to conventional Zn electrolysis. They can be operated at much lower energy consumption, cheaper anode materials can be used, and wastewater can be treated simultaneously. Furthermore, several studies have focused on the bioelectrochemical treatment and purification of acid mine drainage consisting of metal-rich solutions formed by the oxidative dissolution of sulfide minerals exposed to air, humidity, and acidophilic microbes during mining.

Recently, the reduction of Cu^2+^ to Cu^0^ from acid mine drainage in MFC mode, and also Fe, Ni, and tin (Sn) in subsequent MEC operation by applying a cathode potential of −0.7 V vs. Ag/AgCl, has been demonstrated (Leon-Fernandez et al., 2021). In another study, the complete precipitation of aluminum (Al), Zn, Cu, arsenic (As), Cr and almost complete precipitation of Fe, magnesium (Mg), calcium (Ca), manganese (Mn), Ni, Co, lead (Pb), and cadmium (Cd) was achieved using a two-cell bioelectrochemical process with continuous feed (Pozo et al., 2017). Furthermore, high sulfate and Zn concentrations were removed from acid wastewater by conversion of dissolved Zn ions into insoluble zinc sulfide and zinc hydroxide using an acidophilic and autotropic biocathode dominated by *Desulfovibrio* spp. (Teng et al., 2016).

However, no study has investigated the recovery of Zn from metal-laden bioleachate using a MEC. This study aimed to show the potential of combining both processes (bioleaching and bioelectrochemistry) to create a fully biotechnological metal recovery process. Furthermore, the Zn recovery efficiency from bioleachate as a catholyte was compared to the Zn recovery efficiency from the control zinc sulfate solution and the energy consumption under both conditions was evaluated. Furthermore, the anodic composition of the MEC was characterized after three months of operation. Additionally, selective metal precipitation of the bioleachate was investigated, and base consumption was monitored.

## Methods

### MEC setup

The experiments were performed in a two-chamber H-cell separated by a pretreated proton exchange membrane, as described elsewhere (Spiess et al., 2021). The working volume of each chamber was 220 mL. Carbon felt (projected surface area 12.5 cm^2^, AlfaAesar, Heysham, United Kingdom) was pretreated with isopropanol and hydrogen peroxide and used as anode material, as previously described (Spiess et al., 2021). Graphite foil (projected surface area 12.5 cm^2^, 99.8%, AlfaAesar, Heysham, United Kingdom) was used as cathode material, and titanium wires (0.25 mm, Alfa Aesar, Heysham, United Kingdom) were used to enable the external electrical connection. Each chamber was equipped with an Ag/AgCl reference electrode (3 M NaCl, +209 mV vs. SHE).

The anode chamber was filled with 200 mL of nutrient medium (pH 7.2) consisting of the following components (per liter): 3 g KH_2_PO_4_, 2.5 g K_2_HPO_4_, 0.13 g NaCl, 0.31 g NH_4_Cl, 6 g NaHCO_3_, 0.04 g MgSO_4_·7H_2_O, 12.5 mL trace element solution SL 10 (DSMZ 320), and 5 mL vitamin solution (DSMZ 141). Carbon sources with a chemical oxygen demand (COD) concentration of 675 mg L^−1^ were added to simulate wastewater consisting of the following ingredients (per liter): 0.138 g peptone/trypsin, 0.075 g yeast extract, 0.088 g sodium acetate, and 0.37 g glucose monohydrate. The nutrient medium thus prepared is hereafter referred to as synthetic wastewater. Further, the anode chamber was inoculated with 20 mL sewage sludge from a wastewater treatment plant, from which solid particles were first removed by centrifugation at 2,150 g for 10 min. The anode chamber was maintained under anaerobic conditions by flushing with pure CO_2_ at the beginning and after each feeding. A potential of −100 mV vs. Ag/AgCl (3 M NaCl) was applied at the anode using a SP-150 potentiostat (BioLogic Sciences Instruments, Seyssinet-Pariset, France).

The cathode chamber was filled with 220 mL of 36 mM phosphate buffer (pH 7.2) and a stable current flow was observed after 2 months. The cathode potential was monitored regularly with a Voltcraft VC880 multimeter (Hirschau, Germany). During the two-month adaptation period, the anode chamber was supplied with synthetic wastewater twice a week so that 50% of the anolyte and 100% of the catholyte were replaced at each feeding. An adaptation period was required to acclimate microorganisms on MEC conditions and lasted until a stable current flow was observed. All experiments were performed at room temperature with constant stirring of the anolyte and catholyte at 70 rpm with an IKA® RCT basic magnetic stirrer (Staufen, Germany).

### MEC operation

**Figure 1:**
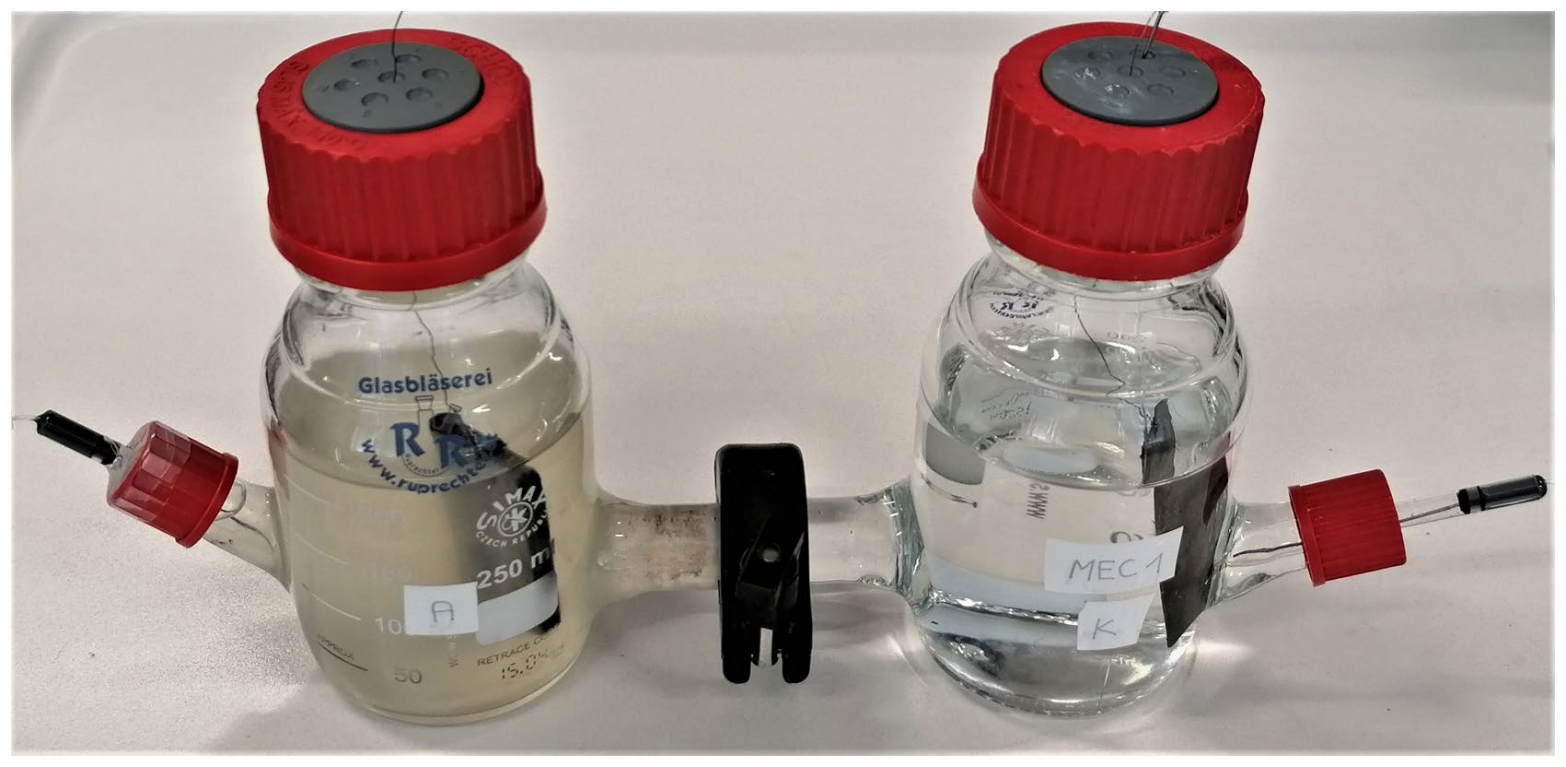
Laboratory setup of the MEC for Zn recovery. A bioanodic chamber, oxidizing synthetic wastewater (left) and generating electrons, coupled to a cathode chamber, reducing metals from the bioleachate (right).

After the adaptation period, the cathode solution was replaced with 220 mL of 7.65 mM zinc sulfate heptahydrate (corresponding to 0.5 g L^−1^ Zn^2+^) with pH adjusted to 3.0 by 1 M sulfuric acid. The electric conductivity of the control zinc sulfate solution thus prepared was 1.6 mS cm^−1^. MEC feeding took place twice a week. At each feeding, 50% of the anolyte was replaced by fresh synthetic wastewater. Simultaneously 100% of the catholyte was exchanged by a new zinc sulfate solution, and the graphite electrode was renewed by removing Zn depositions with 2 M HCl. Before each feeding, samples were collected from the anode and cathode chambers for further analysis. After 12 cycles, 220 mL of diluted filter-sterilized bioleachate was added to the cathode chamber instead of the zinc sulfate solution, and the experiments continued under the same conditions. Prior to use, the bioleachate was filtrated through a Nalgene bottle-top filter with a polyethersulfone membrane (Thermo Fisher Scientific, Waltham, United States) with a pore size of 0.22 μm. Afterwards, the filter-sterilized bioleachate (pH 3) was diluted with deionized water to achieve the same Zn concentrations as the zinc sulfate solution. After dilution, the filter-sterilized bioleachate had a pH of 3.4. Figure 1 depicts the laboratory setup of the MEC for Zn recovery operated in batch mode. The cathode chamber (on the right) was filled with filter-sterilized bioleachate.

### Indirect bioleaching of blast furnace dust

The experiments were carried out as an indirect bioleaching approach. In the first step, the microorganisms *Acidithiobacillus thiooxidans* (DSM 504) and *Acidithiobacillus ferrooxidans* (DSM 14882^T^) purchased from the German Collection of Microorganisms and Cell Cultures (DSMZ, Braunschweig, Germany) were cultivated in basal salts containing 10 g L^−1^ elemental sulfur as previously described (Wakeman et al., 2008) to produce sulfuric acid. In the second step, the produced biogenic sulfuric acid was filter-sterilized and used as a leaching agent to treat blast furnace dust from steel industry. Both steps were conducted at 30°C, 160 rpm stirring speed, and 75 L h^−1^ aeration. During indirect leaching, the average measured redox potential was 638 mV vs. SHE.

### Selective precipitation

Selective precipitation was performed in duplicate by adjusting the pH to 4, 5, 6, 7, 8, and 9. The pH values were determined by a 765 Laboratory pH Meter control (Knick Elektronische Messgeräte GmbH & Co. KG, Berlin, Germany). For each pH value, 25 mL of undiluted bioleachate (pH 3) was continuously stirred using an IKA® C-MAG HS7 plate stirrer (Staufen, Germany) while 1 M NaOH was added until the desired pH was reached. Metal concentrations were determined by ICP-MS in the filtrates (0.22 μm) after six-day precipitation.

### Analytics and calculations

A COD test was used to determine the amount of degraded organic compounds, and then the COD removal efficiency and anodic coulombic efficiency (*CE_An_*) were calculated as described elsewhere (Spiess et al., 2021).

The Zn recovery efficiency was calculated according to Equation (1), where *Zn_1_* is the Zn concentration in the catholyte before electrolysis, and *Zn_2_* is the Zn concentration in the catholyte after electrolysis.


(1) $$Zn\;\mathrm{recovery efficiency}=\frac{\left(Zn_{1}-Zn_{2}\right)}{Zn_{1}}\times 100\%$$

Further, the coulombic efficiency of the cathode (*CE_Zn_*) was calculated according to Equation (2), where *ΔZn* is the recovered amount of Zn (mol), *n* is the number of electrons needed for the reduction of Zn^2+^ to Zn^0^ (2 e^−^), *F* is the Faradays constant (96485.3 C mol ^−1^), *I* represents the recorded current, and *t* is the time.


(2) $$CE_{Zn}=\frac{\Delta Zn\times n\times F}{\int_{0}^{t}I\times dt}\times 100\%$$

The energy consumption (*ηE*) (kWh kg^−1^ Zn) was calculated according to Equation (3), where *E_Cell_* is the cell potential (V), and *M* is the molar mass of Zn (65.38 g mol ^−1^).


(3) $$\eta E=\frac{\int_{0}^{t}\left(E_{Cell}\times I\right)\times dt}{n\times M\times 3600}\times 100\%$$

The energy consumption for COD removal (kWh kg^−1^ COD) was calculated for standard conditions, as previously described (Zeppilli et al., 2019). Statistical analysis was performed using a *t*-test.

### ICP-MS analysis

The metal concentrations in liquid samples were measured using an inductively coupled plasma mass spectrometer Agilent 7,900 (Agilent Technologies, Santa Clara, United States), as previously described (Kremser et al., 2021). Briefly, all samples were filter-sterilized and diluted with Milli Q water by a factor of 100 to minimize the matrix effect and to get the best LOD. A solution of Sc (400 g L^−1^) was used as an internal standard. A set of calibration solutions was prepared for quantification.

### Speciation of dissolved Fe

For measuring Fe^2+^ and total Fe, a microplate reader (Infinite 200 PRO, TECAN, Männedorf, Switzerland) using ferrozine solution was used. Prior to the measurements, samples were diluted as required using distilled water at pH 2. First, 228 μL of ferrozine solution was pipetted into a well of a Microplate 96 well-plate (Greiner, Kremsmünster, Austria), then 12 μL of sample was added. After 10 s of shaking, the first photometric measurements were conducted at 562 nm. Hereafter, 45 μL of hydroxylamine hydrochloride solution and 15 μL ammonium acetate buffer was added. After 20 min, the photometric measurement was repeated. Each measurement was conducted as a triplicate.

### DNA extraction and 16S rRNA gene amplicon sequencing

The bacterial DNA was isolated using the QIAamp BiOstic Bacteremia DNA Kit (Qiagen, Hilden, Germany), according to the manufacturer’s instructions. The highly variable V4 region was amplified with unique barcode primers and sequenced as described previously (Spiess et al., 2021). Briefly, PCR amplification was performed using Platinum II Taq Hot-Start DNA Polymerase (Thermo Fisher Scientific, Waltham, United States), as follows: initial DNA denaturation step at 94°C for 3 min, 35 cycles of DNA denaturation at 94°C for 45 s, annealing at 52°C for 60 s with a 50% thermal ramp, extension at 72°C for 90 s, and a final extension step at 72°C for 10 min. The library was purified by AMPure XP beads (Beckman Coulter, Brea, United States) and sequenced using a MiniSeq System (Illumina, San Diego, United States) with MiniSeq Mid Output Kit (300 cycles). Raw fastq reads were processed in R software (v4.2.2) using the open-source package DADA2 (v1.26) (Callahan et al., 2016). The DECIPHER package was used for multiple alignments with the phangorn package to build a phylogenetic tree, and the phyloseq package was used for subsequent phylogenetic analysis (Vítěz et al., 2020). A summary of all amplicon sequence variants (ASVs) is shown in the Supplementary material. The dataset generated and analyzed in this study is available in the NCBI Sequence Read Archive under project number BioProject ID: PRJNA950388.

## Results and discussion

### Characterization of the bioleachate

Table 1 presents the metal composition of the diluted filter-sterilized bioleachate as prepared for further MEC usage. The bioleachate had the highest metal concentrations of Zn, followed by Al, Fe, Mg, and Mn. Furthermore, the measured electric conductivity of the diluted bioleachate was 3.8 mS cm^−1^ and the pH was 3.4.

**Table 1:** Metal composition of the diluted filter-sterilized bioleachate.

Metals	mg L^−1^ ± SD
Al	270 ± 0.7
Fe	155 ± 0.8
Mg	164 ± 0.6
Mn	48 ± 0.0
Zn	444 ± 0.7

Mean values with standard deviation (SD).

A previous study investigated the thermophilic bioleaching of basic oxygen furnace dust with *Acidianus manzaensis* at an operation temperature of 64°C (Kölbl et al., 2022). Similar to the metal composition results from the bioleachate under mesophilic conditions in Table 1, the previous study reported high metal concentrations of Fe, Zn, Mn, and Al for the leached solution by *A. manzaensis*. These findings demonstrate the great potential of bioleaching and metal recovery from secondary waste products such as basic oxygen furnace dust or blast furnace dust.

### Comparison of Zn recovery from zinc sulfate solution and bioleachate

**Figure 2:**
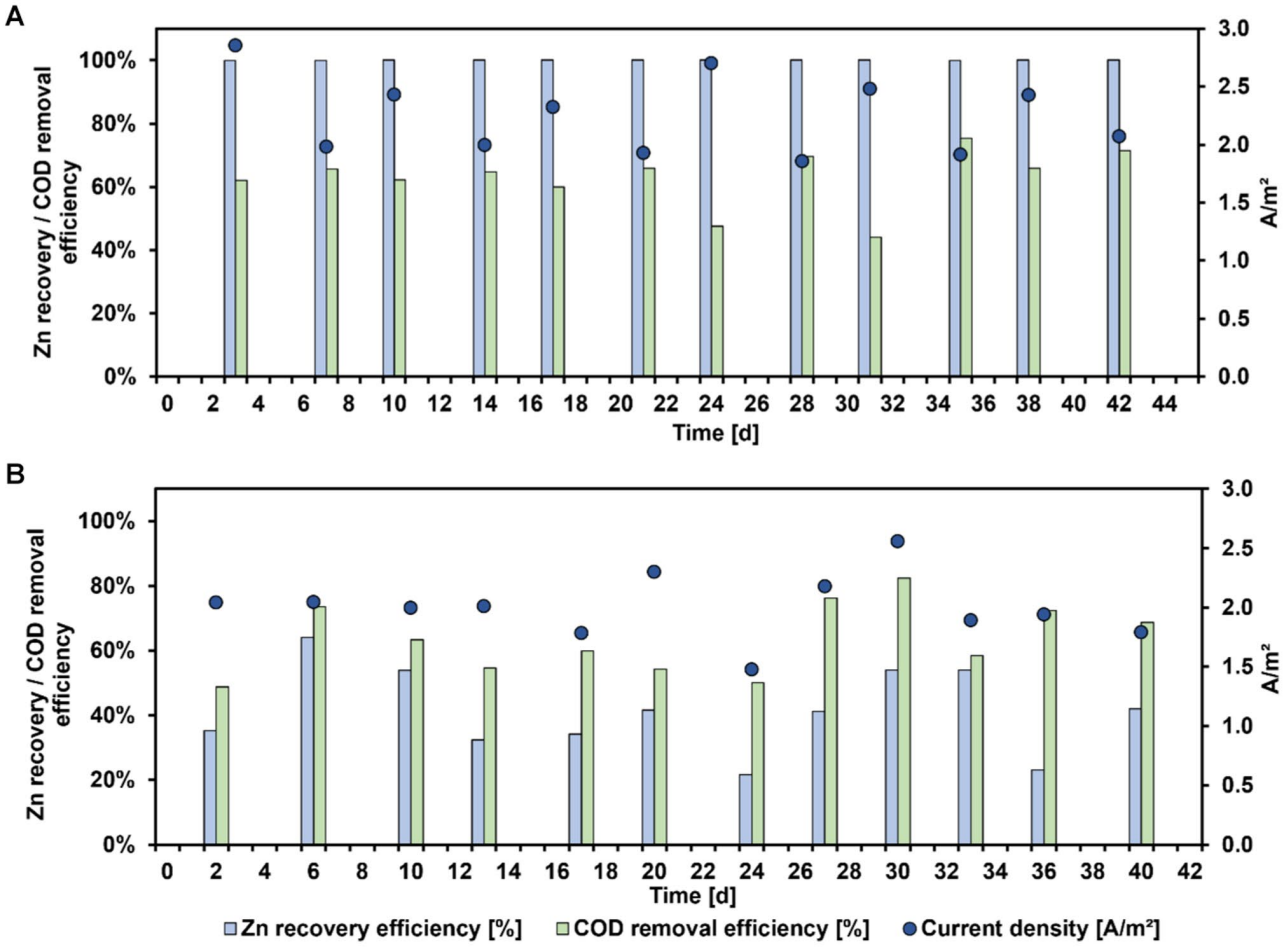
Zn recovery efficiency (blue bars), COD removal efficiency (green bars), and current density (dark blue dots) versus MEC operating time with zinc sulfate solution **(A)** or bioleachate **(B)**.

Figure 2 shows the results of the Zn recovery from zinc sulfate solution (A) and bioleachate (B) over operation time using a MEC. All MEC performance parameters during these experiments are summarized in Table 2. With zinc sulfate solution 100% Zn recovery efficiency at all sampling points was achieved. In comparison, when bioleachate was used as catholyte for the MEC, Zn recovery efficiency fluctuated between 22 and 64%, averaging 41 ± 13%. This difference between the Zn recovery efficiency of zinc sulfate solution and bioleachate was significant (*p* < 0.001). Therefore, the *CE_Zn_* was significantly lower with bioleachate (16 ± 3%) than with the zinc sulfate solution (39 ± 2%) (*p* < 0.001). However, the COD removal efficiency of the bioanode was not significantly different at both conditions, whether using zinc sulfate solution (63 ± 9%) or bioleachate (64 ± 11%) (*p* > 0.05). Also, the difference between zinc sulfate solution (2.2 ± 0.3 A m^−2^) and bioleachate (2.0 ± 0.3 A m^−2^) in current density per projected electrode surface area was insignificant (*p* > 0.05). As the COD removal efficiency remained stable under both conditions, the difference in calculated *CE_An_* was insignificant for the zinc sulfate solution (76 ± 13%) compared to bioleachate (67 ± 10%) (*p* > 0.05).

**Table 2:** Monitored performance parameters of MEC operated with zinc sulfate solution or bioleachate.

Parameters	Zinc sulfate solution	Bioleachate
	Mean ± SD	Mean ± SD
COD removal efficiency [%]	63 ± 9	64 ± 11
Current per m^2^ projected electrode surface [A m^−2^]	2.2 ± 0.3	2.0 ± 0.3
CE_An_ [%]	76 ± 13	67 ± 10
Zn recovery efficiency [%]	100 ± 0	41 ± 13
CE_Zn_ [%]	39 ± 2	16 ± 3

Mean values with standard deviation (SD).

In a previous study, 98.5% Cu was removed from fly ash leachate using a MFC, and in the second step 95.4% Zn and 98.1% Pb were removed using an electrolysis reactor (Tao et al., 2014). In addition to the higher Zn recovery efficiency compared to this study, the reduction time was also faster (95.4% Zn removal in 10 h). However, in the previous study, a voltage of 6 V was applied to empower the recovery of Zn with an electrolysis reactor, which resulted in a high energy consumption of 450 kWh kg^−1^ metal. The present study required an energy consumption of 0.7 kWh kg^−1^ and 2.55 kWh kg^−1^ to recover Zn from zinc sulfate solution and bioleachate, respectively. Therefore, MECs may present a way to recover Zn from leachates at lower energy demands.

**Figure 3:**
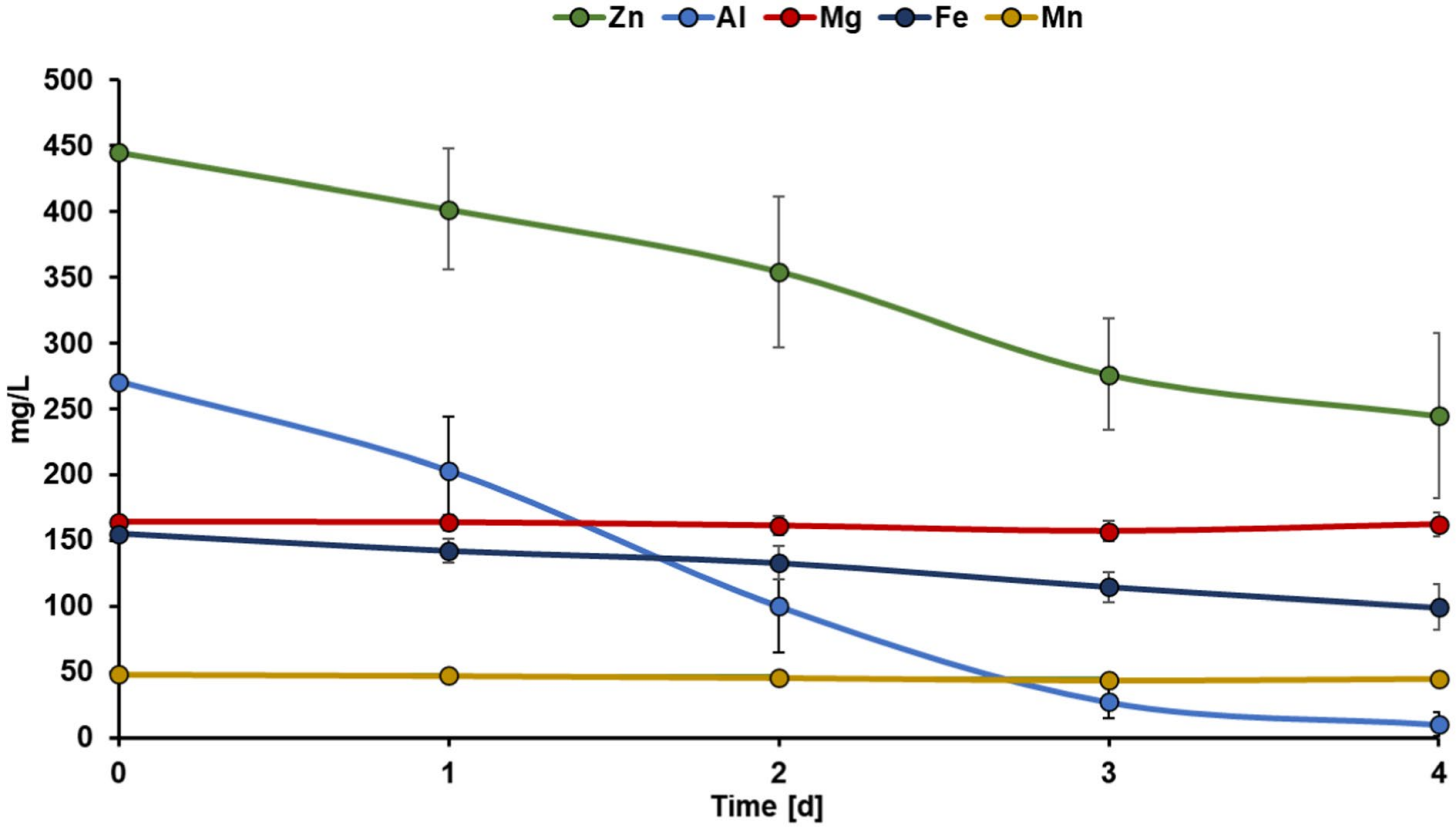
Trends in the concentration of Zn, Al, Mg, Fe, and Mn in the bioleachate used as catholyte over 4 days of MEC operation. Error bars indicate the standard deviation (*n* = 6).

Summarizing, the Zn recovery efficiencies from bioleachate were approximately 60% lower than from the zinc sulfate solution. However, it must be considered that bioleachate has a more complex metal composition compared to the zinc sulfate solution. Therefore, the co-recovery of other metals in the bioleachate was also investigated. Figure 3 shows the concentration trends of the five most abundant metals in the bioleachate during MEC operation. Mg and Mn concentrations remained stable in bioleachate at 162 mg L^−1^ and 45 mg L^−1^ (day 4), respectively, and were not recovered during MEC operation. On the other hand, the concentrations of Zn, Al, and Fe in the catholyte decreased during MEC operation. The Zn concentration decreased continuously from 444 mg L^−1^ (day 0) to 354 mg L^−1^ (day 2) and finally to 245 mg L^−1^ (day 4). The Al concentration decreased rapidly from 270 mg L^−1^ (day 0) to 100 mg L^−1^ (day 2) and further to 10 mg L^−1^ (day 4). The Fe concentration decreased much slower than Zn and Al. After four days, 99 mg L^−1^ Fe from the initial 155 mg L^−1^ remained in bioleachate.

The decrease in Fe concentration in the bioleachate could be due to metal reduction at the cathode. The reduction potential of Fe^2+^/Fe^0^ is −0.45 V vs. SHE, which is below the reduction potential of −0.76 V vs. SHE for Zn^2+^/Zn^0^. Recently, the reduction of Fe^3+^ to Fe^2+^ from acid mine drainage in MFC mode and the subsequent reduction of Fe^2+^ to Fe^0^ deposited on the cathode in MEC mode has been reported (Leon-Fernandez et al., 2021). The reduction of Fe^3+^ to Fe^2+^ took place spontaneously in the first stage (standard reduction potential 0.77 V vs. SHE), but to recover Fe in its elemental form, a potential of −0.7 V vs. Ag/AgCl was applied on the cathode, which led to a 60% Fe decrease after three days of operation (Leon-Fernandez et al., 2021). Similarly, in our experiments, a decrease in the Fe concentration of the bioleachate was observed. Therefore, Fe^2+^ was likely reduced to Fe^0^ at the cathode. On the other hand, the decrease in Al concentration in the bioleachate could be explained by precipitation mechanisms. Al^3+^ possibly precipitated to aluminum hydroxide as the pH increased from 3.4 to 4.4 during the electrolysis. These assumptions are consistent with previously published results (Wei et al., 2005) showing >75% Al precipitation from acid mine drainage when the pH rose from 3.5 to 4.5.

**Figure 4:**
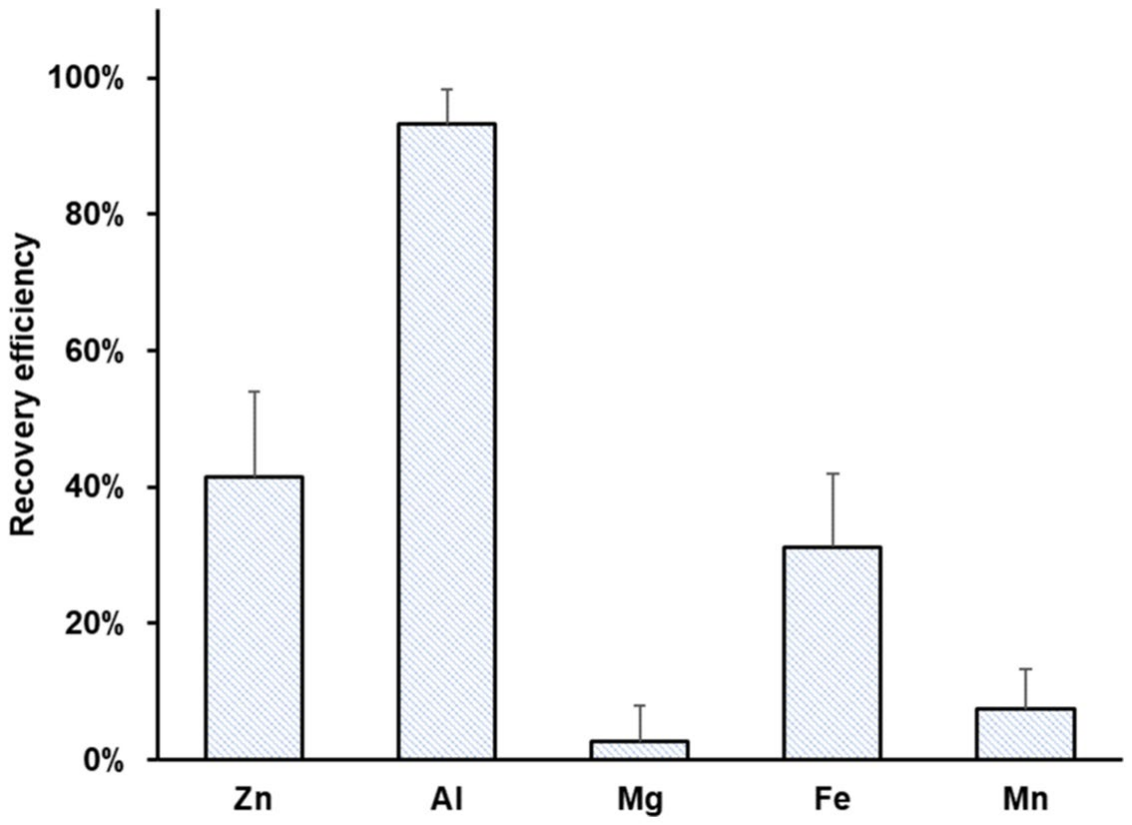
Average recovery efficiency of Zn, Al, Mg, Fe, and Mn using bioleachate as catholyte in MEC operation. Error bars indicate the standard deviation (*n* = 12).

Figure 4 summarizes the recovery efficiencies of Zn, Al, Mg, Fe, and Mn. Al showed the highest recovery efficiency (93 ± 5%), possibly due to the precipitation of aluminum hydroxide. Moderate recovery efficiencies were achieved for Zn (41 ± 13%) and Fe (31 ± 11%) due to a reduction at the cathode. Mn and Mg were recovered with minimal efficiency, 3 ± 5 and 7 ± 6%, respectively.

The Zn recovery from bioleachate was substantially lower than from zinc sulfate solution because the provided electrons were probably used for competing reactions such as Fe^2+^/Fe^0^ reduction. The MEC operation time probably needs to be expanded to increase the efficiency of Zn recovery from bioleachate. As observed earlier, the recovery time depends on the initial concentration and the applied current (Lim et al., 2021).

### Energetic evaluation

Table 3 summarizes the average energetic parameters from Zn recovery experiments using zinc sulfate solution or bioleachate of this study compared to literature. The difference between the energy consumptions for Zn recovery from zinc sulfate solution (0.70 kWh kg^−1^) and bioleachate (2.55 kWh kg^−1^) was significant (*p* < 0.001) due to the higher amount of Zn recovered from zinc sulfate solution than bioleachate. In addition, the difference between the average cell voltages (E_Cell_) using zinc sulfate solution (−1.01 V) and bioleachate (−1.39 V) as the catholytes was significant (*p* < 0.05). On the contrary, the difference between the energy consumption for COD removal (0.20 and 0.22 kWh kg^−1^ COD) was insignificant (*p* > 0.05).

**Table 3:** An overview of the MEC energy parameters of this study compared with literature.

Parameters	This study		Modin et al. (2017)	Ramachandran et al. (2004)
	Zinc sulfate solution	Bioleachate		
Zn [mg L^−1^]	500	444	413	55.000
E_Cell_ [V]	−1.01	−1.39	−1.24	−3.26
kWh kg^−1^ Zn	0.70	2.55	2.4	3.51
kWh kg^−1^ COD	0.20	0.22	n.d.	n.d.

The calculated energy consumption of 0.7 kWh kg^−1^ Zn from zinc sulfate solution was lower compared to 2.4 kWh kg^−1^ of a previous study (Modin et al., 2017). Similar to this study, the MEC has been previously operated with controlled anode potential, and ZnSO_4_ served as the source for Zn^2+^. However, the lower amount of Zn recovered using bioleachate resulted in a higher energy consumption of 2.55 kWh kg^−1^ Zn. In another study, the electrowinning of Zn from zinc ash leachate was investigated with a PVC cell with an applied current density of 200 A m^−2^, consisting of an aluminum cathode and an iridium dioxide anode. The zinc-sulfate electrolyte was circulated, and Zn was stripped every 24 h. Compared to the MEC operated with zinc sulfate solution in this study, a five times higher energy consumption (3.51 kWh kg^−1^) was required to recover Zn with electrowinning (Ramachandran et al., 2004). The higher energy consumption for Zn electrowinning compared to the lower energy consumption using a MEC with zinc sulfate solution (0.70 kWh kg^−1^) or bioleachate (2.55 kWh kg^−1^) in this study profiles bioelectrochemical Zn recovery as a desirable candidate compared to electrowinning.

### Metagenomic analysis

**Figure 5:**
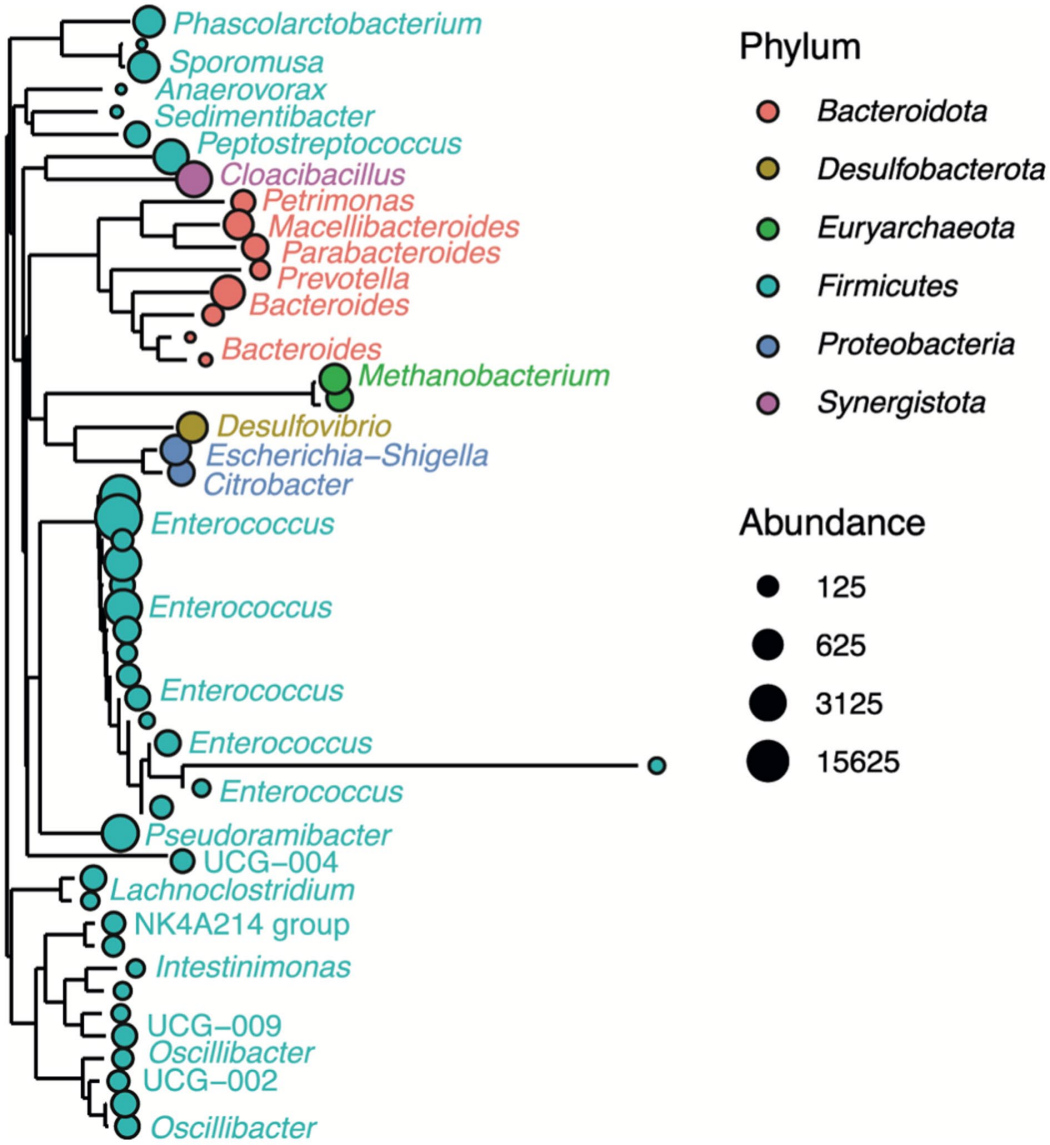
Phylogenetic tree showing the genetic relationships of the anodic microbial community in the MEC. Only the 50 most abundant representatives are shown. The circle color represents phylum taxa, and the circle size corresponds to their abundance (number of reads). The tree is labeled by color-coded representatives identified in the taxa of the genus.

Figure 5 shows the genetic relationships of the anodic microbial community after three months of MEC operation, and Table 4 summarizes the relative abundances of the microbial community composition enriched on the MEC anode. The genus *Enterococcus* strongly dominated the anodic biofilm with a relative abundance of over 80%. The other four representatives were below 3%. Over 10% were represented by unknown genera or fell below the 1% threshold. The species diversity values obtained are similar to those previously observed under the same experimental conditions (Spiess et al., 2021). *E. faecalis* is an electroactive Gram-positive bacterium commonly identified on MFC anodes, capable of direct and mediated electron transport (Pankratova et al., 2018). *E. faecalis* forms dense biofilms in the presence of glucose (Pillai et al., 2004). Since the main carbon source in the synthetic wastewater in this study was glucose, this may explain why *Enterococcus* dominated the anode after the enrichment from sewage sludge. Similar observations were made when using glucose-rich synthetic wastewater as a substrate in a fully biocatalyzed MEC for methane production (Spiess et al., 2021).

**Table 4:** Microbial community composition enriched on the anode surface in the MEC.

Genus	Relative abundance (%)
*Enterococcus*	81.45
*Pseudoramibacter*	2.77
*Cloacibacillus*	2.10
*Acetobacterium*	1.73
*Bacteroides*	1.20
Others	10.76

Obs (169), Cha (233), Sha (1.7), InS (2.1).

Only representatives of genera with relative abundance higher than 1% are shown. Alpha diversity was estimated using the following indices: Observed (Obs), Chao1 (Cha), Shannon (Sha), and Inverse Simpson (InS).

### Selective metal precipitation

**Figure 6:**
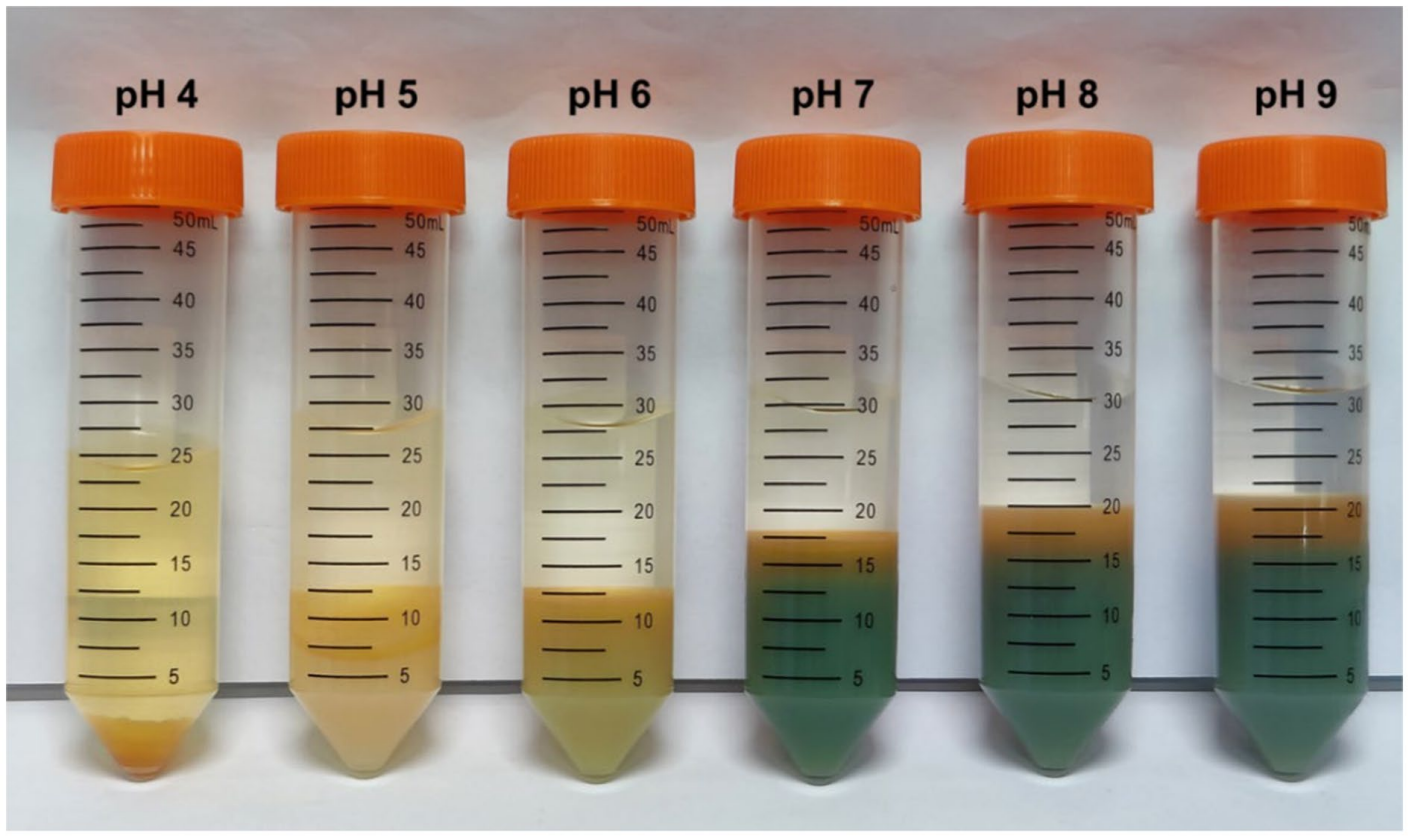
Selective precipitation of metals in the pH range between 4 and 9.

Furthermore, selective metal precipitation from the bioleachate was performed to investigate possible precipitation mechanisms due to the catholyte alkalization during MEC operation. For instance, Al^3+^ likely has been precipitated as aluminum hydroxide when the catholyte pH increased from 3.4 to 4.4 during MEC operation. Figure 6 shows tubes filled with bioleachate after performing selective precipitation at different pH values. As the pH grew, the proportion of precipitation products increased while the liquid fraction decreased. Furthermore, a color change was noticeable from pH 7, probably caused by the precipitation of Fe and Mn.

**Figure 7:**
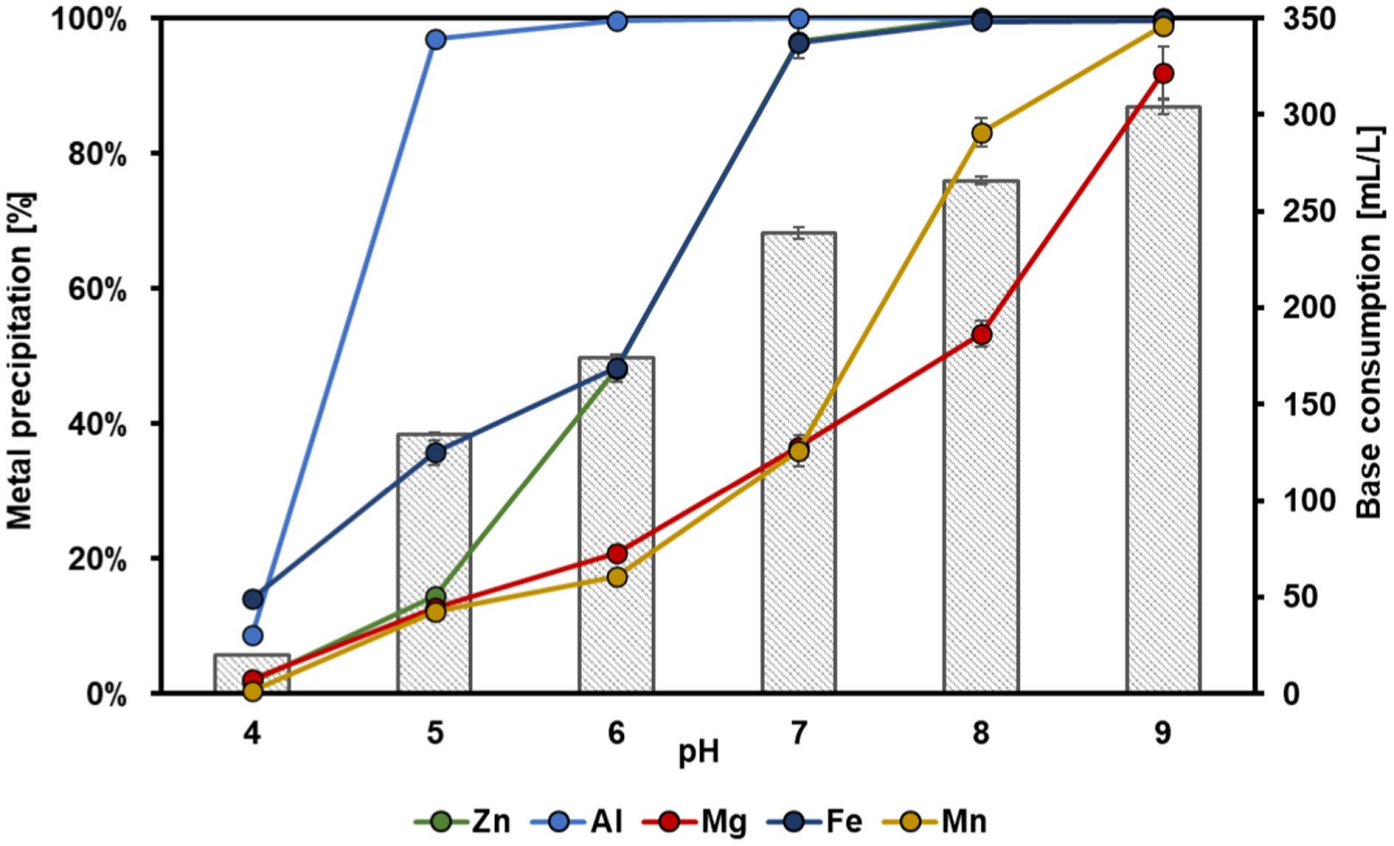
Dependence of metal precipitation percentage on different pH and base consumption. The five metals with the highest concentrations from Zn, the most abundant one, up to Mn, are shown. Error bars indicate the standard deviation (*n* = 2).

The dependence between the most precipitated metals and the base consumption to adjust different pH values in the bioleachate is shown in Figure 7. Fe (14 ± 0.6%) and Al (9 ± 0.5%) started precipitating at pH 4. Almost all Al was precipitated (97 ± 0.1%) with a base consumption of 135 ± 0.6 mL L^−1^ at pH 5, whereas Zn, Mg, and Mn remained largely in solution (<15% precipitation). This supports our findings that Al^3+^ probably precipitated as aluminum hydroxide during Zn electrolysis as the pH in the catholyte increased from pH 3.4 to 4.4. Hence, the selective recovery of Al from bioleachate at pH 5 before its filling into the MEC cathode chamber could be used to increase the selectivity of the bioelectrochemical Zn recovery. A further pH increase to pH 6 resulted in the precipitation of 48 ± 1.9% Zn, 100 ± 0.1% Al, 21 ± 1.0% Mg, 48 ± 0.5% Fe and 17 ± 0.7% Mn. Zn and Fe showed an almost simultaneous trend in precipitation from pH 6 to 9. At pH 7, almost all Zn (97 ± 1.0%) and Fe (96 ± 2.4%) were precipitated at a base consumption of 239 ± 3 mL L^−1^. In contrast, only 37 ± 0.3% Mg and 36 ± 2.3% Mn were precipitated. At pH 8, Zn and Fe precipitated 100%, and a further increase in pH to 9 with a total base consumption of 304 ± 4 mL L^−1^ resulted in precipitation of 92 ± 3.8% Mg and 99 ± 0.5% Mn.

The selective precipitation experiments revealed almost complete Al precipitation at pH 5, whereas the other metals remained widely dissolved. These findings coincide with a previous study in which 97% Al was recovered at pH 5, and Mn and Fe remained nearly stable in the solution (Kremser et al., 2022). However, the addition of base to adjust pH 5 in this study was substantially higher (135 ± 0.6 mL L^−1^ 1 M NaOH) than in the previous one (46.8 mL L^−1^ 1 M NaOH). However, it must be noted that in the previous study, a synthetic metal solution was used instead of bioleachate, which probably had a higher buffer capacity. At pH 7, more than 95% of Zn and Fe precipitated simultaneously, whereas pH 9 is required to precipitate most of Mg and Mn. These results are consistent with the previously described coprecipitation of Zn^2+^ and Fe^2+^ from industrial wastewater at pH 6.5–8.5 (Wang and Chen, 2019). Fe speciation of the used bioleachate in this study revealed a differentiation of 91% Fe^2+^ and 9% Fe^3+^ species. As reported earlier, Fe^3+^ precipitates at pH 3, whereas Fe^2+^ precipitation needs higher pH-values (Kremser et al., 2022), as also observed in this study. This finding was also supported by the dark blue-green color of the precipitate (Figure 6). The precipitation of metal ions can be influenced by various factors such as temperature (Cao et al., 2009) or initial concentrations. Especially the latter is of great importance because the higher the metal ion’s initial concentration, the lower the pH at which it starts to precipitate (Wang and Chen, 2019), which could explain why 48 ± 1.9% Zn was already precipitated at pH 6. Furthermore, sulfide and hydroxide precipitation mechanisms have previously been distinguished by adding NaOH until pH 4 to precipitate Fe selectively from synthetic metal solution (Santaolalla et al., 2021). Subsequently, Na_2_S was added to precipitate Cu, Zn, and Ni at pH 6. However, Cu (20.7%) and Ni (9.2%) have coprecipitated with Fe during the first step (Santaolalla et al., 2021).

To increase the selectivity of Zn recovery from bioleachate using MECs, pre-precipitation of Al (approximately at pH 5) by adding NaOH and subsequent filtration before entering the MEC cathode outlines a possible way. Furthermore, impurities such as Fe could be separated from bioleachate by oxidation of Fe^2+^ to Fe^3+^ using H_2_O_2_ (Wang and Chen, 2019) or biooxidation (Nurmi et al., 2010), followed by Fe^3+^ precipitation at pH 3 prior to bioelectrochemical Zn recovery. Therefore, to increase the selectivity of metal recovery from bioleachate, a combination of selective precipitation of leachate impurities, such as Al and Fe, with the subsequent operation of a Zn recovering MEC, must be further investigated and carefully evaluated for potential application. In addition, the composition of precipitates and purity of deposited cathode metals should be determined in the future.

## Conclusion

This study investigated the recovery of Zn from bioleachate using a MEC for the first time. During Zn recovery, a constant potential of −100 mV vs. Ag/AgCl was applied at a synthetic wastewater-treating bioanode. First, the recovery of Zn from the control zinc sulfate solution and then from bioleachate was examined. Despite decreased Zn concentration from 444 mg L^−1^ to 245 mg L^−1^ during MEC operation with bioleachate, the Al concentration dropped from 270 mg L^−1^ to 10 mg L^−1^ after four days of operation. The drop in Al concentration was probably due to the precipitation of aluminum hydroxide because of an increase in the catholyte pH from 3.4 to 4.4. As the selective precipitation experiments confirmed, Al is precipitated almost completely at pH 5. Furthermore, during Zn recovery from bioleachate, the simultaneous reduction of Fe was observed. Moreover, the energy consumption for a Zn recovering MEC, whether operated with zinc sulfate solution or bioleachate, was substantially lower than electrowinning. In principle, Zn recovery from bioleachate using a MEC is feasible. Still, lower Zn recovery efficiency (41 ± 13%) and energy consumption (2.55 kWh kg^−1^) were achieved compared to zinc sulafate solution due to a more complex composition of the bioleachate. After three months of MEC operation, *Enterococcus* was enriched on the anode with 81.45% from sewage sludge. The predominance of this electroactive species is consistent with MEC operating and growth conditions that include glucose as the primary carbon source.

## Data availability statement

The datasets presented in this study can be found in online repositories. The names of the repository/repositories and accession number(s) can be found in the article/Supplementary material.

## Author contributions

SS: conceptualization, methodology, investigation, visualization, and writing—original draft. JK: data analysis, visualization, and writing—original draft. TV: data acquisition and analysis. LB: providing dust samples and writing—review and editing. CH: investigation. ASC: writing—review and editing. MM: statistical analysis, funding, and writing—review and editing. MH: project administration, supervision, funding, conceptualization, and writing—review and editing. All authors contributed to the article and approved the submitted version.

## Funding

The project was supported by the European fund for regional development, the program Interreg V-A Austria—Czech Republic, project ATCZ291, OPTIMO (Optimierung einer nachhaltigen Schwefelsäureproduktion für (Bio)Leaching-Prozesse im Abfallsektor). The financial support of the Province Styria and the Styrian Business Promotion Agency (“Zukunftsfonds Steiermark”) within the project INNOMET (PN 1507) is gratefully acknowledged. Further, the authors acknowledge the funding support of K1-MET GmbH, metallurgical competence center. K1-MET is a COMET Centre within the COMET – Competence Centers for Excellent Technologies Program and funded by the Federal Ministry for Climate Action, Environment, Energy, Mobility, Innovation and Technology; the Federal Ministry for Labor and Economy; the provinces Upper Austria, Styria and Tyrol; the Styrian Business Promotion Agency, and the Standortagentur Tyrol. This work was supported by a subsidy for the development of the research institution and by the Masaryk University Program, project no. MUNI/A/1313/2022.

## Conflict of interest

SS, CH, ASC, and MH were employed by K1-MET GmbH. LB was employed by Voestalpine Stahl GmbH.

The remaining authors declare that the research was conducted in the absence of any commercial or financial relationships that could be construed as a potential conflict of interest.

## Publisher’s note

All claims expressed in this article are solely those of the authors and do not necessarily represent those of their affiliated organizations, or those of the publisher, the editors and the reviewers. Any product that may be evaluated in this article, or claim that may be made by its manufacturer, is not guaranteed or endorsed by the publisher.
